# Epidemiological Studies on Eye Diseases in Centers for Stray Dogs in Northwestern Romania

**DOI:** 10.3390/vetsci12050480

**Published:** 2025-05-15

**Authors:** Elena Bonea, Cornel Dionisie Igna, Monica Ocnean, Bianca Cornelia Lungu, Ioan Hutu

**Affiliations:** 1 Horia Cernescu Research Unit—Faculty of Veterinary Medicine, University of Life Sciences “Regele Mihai I”, Calea Aradului 119, 300645 Timisoara, Romania; elena.bonea@fmvt.ro (E.B.); corneligna@usvt.ro (C.D.I.); ioan.hutu@fmvt.ro (I.H.); 2 S.C. Mobovet SRL Veterinary Clinic, 3th Ceahlaului Street, 440043 SatuMare, Romania; 3 Faculty of Management and Rural Turism, University of Life Sciences “Regele Mihai I”, Calea Aradului 119, 300645 Timisoara, Romania

**Keywords:** stray dogs, epidemiology, eye diseases, ophthalmic affections

## Abstract

Eye disease in dogs is a common problem that can affect both the health and well-being of the animals. The aim of the study was to investigate the prevalence and incidence of the most common eye diseases in a large population of dogs from two stray dog centers in northwestern Romania over a two-year period.We conducted a retrospective observational study of 208 eye diseases in two dog shelters in northwestern Romania: the “Ham Ham” Association and the “Free Life” Association in Satu Mare County. The study spanned from January 2022 to December 2023. We collected data on eye disease status from a total of 2293 dogs. The dogs were categorized into three age groups: young dogs (under 2 years), adult dogs (2 to 7 years), and senior dogs (over 7 years). Additionally, the dogs were grouped by sterilization status and gender. The statistical analysis was performed using Pearson’s chi-squared test, Student’s *t*-test, and binary logistic regression. The prevalence of ocular diseases in this study was 9.07%, with 208 out of 2293 dogs diagnosed with eye problems. The most common ocular diseases observed were conjunctivitis (58/208 or 27.9%), eyelid issues (50/208 or 24%), cataracts (40/208 or 19.2%), glaucoma (13/208 or 6.3%), keratopathies (10/208 or 4.8%), traumatic eye injuries (10/208 or 4.8%), foreign bodies (7/208 or 3.4%), blepharitis (5/208 or 2.4%), proptosis (3/208 or 1.4%), progressive retinal atrophy ((3/208 or 1.4%), retinal detachment ((3/208 or 1.4%), and dry eye (2/208 or 1%). Among the three age groups, puppies and young dogs (under 2 years) were the most affected, with a punctual prevalence of 50.96% at the “Free Life” center and 65.51% at the “Ham Ham” center. Compared with older dogs, the younger dogs were 4.91 times more likely to develop conjunctivitis (OR = 4.91, *p* = 0.001), 9.38 times more likely to develop eyelid problems (OR = 9.38, *p* < 0.000), and 0.31 times less likely to develop cataracts (OR = 0.31, *p* = 0.019).Our epidemiological study found that eye diseases are common among shelter dogs, with incidence rates (new cases over a specific period) varying by age. The youngest and oldest dogs were the most affected, with the highest prevalence observed in the youngest group. The study also highlighted the significant impact of factors such as the shelter center (*p* < 0.000), age (*p* < 0.000), and sterilization status (*p* < 0.000) on the prevalence of eye disorders. These findings underscore the need for enhanced awareness about eye diseases and their risk factors to facilitate early diagnosis and treatment.

## Simple Summary

The study aimed to identify the epidemiological situation of eye diseases in dogs in a timely manner from two stray dog centers in Northwestern Romania. Eye disease in dogs is a common problem that may affect both the health and well-being of the animals. In this study, we evaluated the occurrence and incidence of the most common eye diseases and their impact on general life quality in the canine population from the two dog shelters over a 2-year period. The study traces stray dogs of all ages, which we divided into three categories: young, adult, and old dogs. The most common ocular diseases were conjunctivitis, eyelid problems (entropion, ectropion, cherry eye), cataracts, glaucoma, keratopathies, keratoconjunctivitis sicca, eyeball trauma, ocular foreign bodies, and eye proptosis. This study indicates that eye diseaseincidenceis high in shelter dogs and that it varies depending on the dogs’ age and sterilization status. Thus, information about eye diseases andrisks should be kept under surveillance to promote early diagnosis and treatment in order to prevent the effect of visual impairment on quality of life and the increase in care costs as a result of complications of primary eye diseases, as well as to increase the adoption rate for shelter dogs with eye diseases.

## 1. Introduction

The eye is a unique and highly complex organ in terms of structure and function; it should never be viewed or examined in isolation but rather as an integral part of the entire body, as numerous systemic diseases manifest in the eyes. Its sensitivity means that even mild disturbances to its homeostasis, whether from direct injury or other local or systemic conditions, can significantly impact its function. Accurate diagnosis in veterinary ophthalmology often relies on anatomical observations and various straightforward ophthalmologic techniques [1].

There is an abundance of literature on eye conditions in dogs commonly encountered by practitioners [1,2,3,4,5]. There is significant interest in hereditary eye diseases and their prevention among veterinarians and breeders, as inherited eye disorders are more common in dogs than in any other domestic species [2]. However, preventing inherited eye diseases in stray dogs, which are often crossbreeds, is challenging due to their unknown histories. In stray dog centers, dogs with ocular diseases, such as retinal detachment, progressive retinal atrophy, dry eye, glaucoma, and cataracts, often face difficulties being adopted.

Studies have shown that conjunctivitis is the most frequently diagnosed clinical condition in dogs, with the eyelid and conjunctiva being the most affected anatomical locations [3,4,5]. Other common ocular conditions reported include pigmentary keratitis and corneal ulcers/eye injuries [4]. Regarding ocular affections and age, most studies concluded that the majority of ocular conditions occur in dogs less than 5 years of age [3]. Some authors [3,6] reported a higher percentage of ophthalmic problems in dogs less than 5 years of age, while other studies [7,8] indicated that age-related eye conditions are more prevalent in older dogs. The highest incidence of ocular affections has been recorded in non-descript breeds, with maximum cases in the young age group [4]. However, some studies present contradictory findings about age as a risk factor. In Europe and Romania studies, various etiological factors, including infectious [9,10], parasitological [11,12], metabolic [13,14], and toxic exposures [15], have been reported to be associated with eye diseases in dogs.

According to our understanding, reports on the incidence, diagnosis, and management of eye affections in stray dogs have not been documented despite the growing number of stray dogs across Romania, both in cities and the countryside. With the increasing demand for shelters and centers, this work presents a retrospective study aimed at determining the occurrence, incidence risk, point prevalence, and management practices for preventing eye affections in stray dogs in northwest Romania.

## 2. Materials and Methods

### 2.1. Organizing and Area of Study

The epidemiological retrospective study utilized medical records from the stray dog centers “Ham Ham” and “Free Life”, along with their contracted veterinary clinic, “Mobovet SRL”, located in Satu Mare County, northwest Romania. The “Free Life” association center is the largest stray dog center in northwest Romania, with the number of dogs varying between 550 and 650 annually. The administering association is dedicated to sustaining the expenses, protection, and welfare of dogs. The operational costs in both mentioned stray dog centers encompass staff salaries (caretakers, administrative, and maintenance workers), veterinary care (check-ups, vaccinations, treatments, and sterilization), animal supplies (food, bedding, grooming items, and accessories), facility maintenance (utilities, repairs), administrative expenses (office supplies, insurance, and permits), transportation (vehicles, fuel, and maintenance), waste management (disposal and sanitation), and emergency funds for unforeseen medical or repair costs.

Most of the dogs collected by these associations are strays found on city streets, in villages, or in the countryside and forests of northwest Romania. A smaller number of collected dogs are those abandoned by owners due to financial constraints, medical issues, behavioral problems, or lack of time. The “Ham Ham” Association is a new center that opened at the end of 2021. They started with a population of 125 dogs in 2022 to reach a number of 195 dogs at the end of 2023, with a constant increase in this number of dogs. In the “Free Life” center, the number of adoptions varies from 1 to 10 dogs monthly, and in the “Ham Ham” center, it varies from 10 to 20 dogs. The newly admitted dogs are sterilized a maximum of two months after arrival; thus, in order to manage the dog population, all dogs from the centers become reproductive sterile.

### 2.2. Data Retrieved, Inclusion Criteria and Considered Risk Variables

Data Retrieval: Data for this study were collected over a two-year period from January 2022 to December 2023. We reviewed medical records and ophthalmic examination reports for a total of 2293 dogs to identify those with diagnosed ocular diseases. Nearly all dogs in both shelters were mixed breed, with no purebred dogs. Only a few were mixed breeds of retrievers, sheepdogs, dachshunds, Bichon crossbreeds, Shihtzu, or Pekinese. Therefore, breed was not used as a reference criterion for data collection.

Inclusion Criteria were: age—dogs aged 1 month and older were included in the study; diagnosis—only dogs with documented ocular diseases were included; shelter information—dogs from both the “Ham Ham” and “Free Life” shelters were considered; and data completeness—only dogs with complete ophthalmic examination records were included for accurate data analysis.

Considered Risk Variables: The considered risk variables for eye disease included shelter type (C1: “Free Life”, C2: “Ham Ham”), age groups (A1: young, under 2 years; A2: adult, 2–7 years; A3: senior, above 7 years), sex (F: female, M: male), sterilization status (Y: sterilized, N: not sterilized), and clinical examination time points (P1: January 2022, P2: December 2022, P3: December 2023) for splitting the study period into two equal periods. These variables were analyzed to determine their association with the prevalence of ocular diseases and to understand their impact on the epidemiology of eye disorders in the study population.

### 2.3. Ophthalmic Examination

To determine the incidence and distribution pattern of ocular affections, dogs with eye disorders underwent a detailed ophthalmological examination. The examination process (diagnostic method) included the following steps (Table 1):

Special examinations, such as ophthalmoscopy and tonometry, were performed only when specifically required by the dog’s condition. Following the ophthalmic examination (Table 1), it was possible to diagnose the following ocular conditions: blepharitis, cataracts, conjunctivitis, dermatocele, dry eye, eyelid disorders, presence of foreign bodies, glaucoma, keratopathies, lens luxation, congenital microphthalmia, progressive retinal atrophy, proptosis of the eyeball, retinal detachment, traumatic injuries, and unilateral congenital anophthalmia. A total of 208 such cases are detailed in Table 2 and Table 3.

### 2.4. Epidemiological and Statistical Analysis

The occurrence of ocular disorders was determined through the evaluation of case records from the “Mobovet” Veterinary Clinic in Satu Mare for both stray dog centers. Ocular diseases were classified based on (i) the anatomical location of the lesion, (ii) the clinical type of the lesion, and (iii) the method of diagnosis and treatment. Cases were organized by shelter, time of diagnosis, age group, sex, and spay status to calculate the incidence and prevalence of eye disorders.

**Incidence** refers to the number of newly diagnosed cases of a disease that occur in the shelter’s population. The two essential components of an incidence value are (i) the number of new cases and (ii) the period of time over which the new cases occur [16]. **The incidence risk** was calculated as the ratio between the number of new cases identified during one year and the number of dogs at risk at the beginning of that year, multiplied by 100.

**Prevalence** refers to the number of instances of dogs with eye disease existing in the shelter’s population at a designated time without distinguishing between old and new cases. **Point prevalence** refers to the amount of disease in a population at a particular point in January 2022, December 2022, and December 2023. It was calculated as the ratio between the number of existing cases at each time point and the total population at that time multiplied by 100 [16].

In order to observe the association of the factors with eye disease, we used statistical tests such as Pearson’s chi-squared test and student test performed using *SPSS Statistics for Windows*, Version 17.0 (IBM Corp., Chicago, IL, USA, 2008). A multivariable binary logistic regression analysis was performed using Minitab version 14.1 (Minitab Inc., LLC, State College, PA, USA, 2003) to identify factors associated with the presence of ocular disease. The dependent variable was binary (presence or absence of the ocular disease), and the predictors included the variables mentioned in Table 3 (center, age, gender, time, and splayed). A *p*-value < 0.05 was considered statistically significant. The logit link function was used.

## 3. Results and Discussions

During the study period, the stray dog centers “Ham Ham” and “Free Life” had average monthly operational expenses of €27.75 ± 1.01 per dog and €32.13 ± 1.18 per dog, respectively, with a significant difference between them (*p* = 0.007). Overall, these expenses increased gradually from €27.83 ± 1.06 per dog in 2022 to €32.04 ± 1.13 per dog in 2023, a statistically significant rise (*p* = 0.010).

### 3.1. Point Prevalence of Ocular Diseases Along the Study Period

During the study, which covered a total of 2293 dogs across three time points (January 2022 with 675 dogs, December 2022 with 780 dogs, and December 2023 with 838 dogs), 208 cases of ocular affections were recorded. Over the study period, an increase in both the number of dogs in the shelters and the incidence of eye pathologies year over year was observed. The overall prevalence of eye affections during the study period was 9.07%, with a relatively even distribution of annual point prevalence at each measurement point (Table 2). Statistical analysis showed no significant differences in the number of ocular cases over the years (χ^2^ = 0.168, *p* = 0.919) within each shelter (“Free Life” χ^2^ = 2.273, *p* = 0.321 and “Ham Ham” χ^2^ = 3.109, *p* = 0.211), or between the two shelters (χ^2^ = 4.707, *p* = 0.095) despite the differences between the monthly operational expenses.

**Table 2:** Annual prevalence and total prevalence of ocular diseases in dogs in January 2022, December 2022, and 2023.

	Numbers of Dogs in:			Dogs with Ocular Diseases in:				Point Prevalence %
	“Free Life”	“HamHam”	Total	“Free Life”	“HamHam”	Total	Cumulative	
2022 Jan (P1)	550	125	675	43	21	64	64	9.48%
2022 Dec (P2)	620	160	780	36	33	69	133	8.85%
2023 Dec (P3)	645	193	838	51	24	75	208	8.95%
Total	1815	478	2293	130	78	208	208	9.07%

In 2022, 69 new cases of ocular problems were identified (133 − 64), with a population at risk of 611 dogs (675 − 64), resulting in an annual incidence risk of approximately 11.3%. For 2023, 75 new cases were recorded (209 − 133), with a population at risk of 647 dogs (780 − 133), yielding an annual incidence risk of approximately 11.75%. This corresponds to a monthly incidence risk of approximately 0.94% in 2022 and 0.98% in 2023, assuming a uniform distribution of new cases across the stray dog centers. At Ham Ham Center, 33 new ocular cases were identified in 2022 (incidence risk: 31.73%), and 24 in 2023 (22.64%), with corresponding monthly risks of 2.64% and 1.89%. At Free Life Center, 36 new cases were recorded in 2022 (7.10%) and 51 in 2023 (9.42%), with monthly risks of 0.59% and 0.79%, respectively. These values assume a uniform distribution of cases across each year. No statistically significant difference in incidence risk was found between the centers (χ^2^ = 1.210, *p* = 0.271).

### 3.2. Point Prevalence of Common Ocular Diseases

From January 2022 to December 2023, the range of ocular diseases observed in dogs included conjunctivitis, eyelid issues, cataracts, keratopathies, glaucoma, eye trauma, foreign bodies (mainly grass awns and a single case of wood chip), proptosis, blepharitis, dry eye, congenital microphthalmia, unilateral congenital anophthalmia, and retinal detachment (see Table 3).

**Table 3:** The distribution of ocular affections in dogs by center, age, sex, and castration.

The Diagnosis of Ocular Affections in Alphabetical Order Includes:	Total Cases			Number of Dogs with Ocular Disease by:									
		Center		Group Age			Measurement Point			Sex		Spayed	
		C1	C2	A1	A2	A3	P1	P2	P3	F	M	Y	N
1. Blepharitis	5 (2.4%)	5	0	0	4	1	1	2	2	4	1	0	5
2. Cataracts	40 (19.2%)	35	5	0	10	30	14	13	13	19	21	2	38
3. Conjunctivitis	58 (27.9%)	30	28	43	6	9	19	22	17	32	26	33	25
4. Dermatocele	1 (0.5%)	0	1	1	0	0	1	0	0	0	1	1	0
5. Dry eye	2 (1.0%)	2	0	0	2	0	0	0	2	2	0	0	2
6. Eyelids problems *	50 (24.0%)	24	26	35	11	4	18	16	16	26	24	23	27
7. Foreign body	7 (3.4%)	3	4	4	3	0	2	3	2	2	5	2	5
8. Glaucoma	13 (6.3%)	13	0	0	5	8	3	5	5	5	8	0	13
9. Keratopathies **	10 (4.8%)	7	3	1	6	3	2	4	4	7	3	1	9
10. Lens luxation	1 (0.5%)	0	1	0	1	0	1	0	0	0	1	1	0
11. Microphthalmia congenital	1 (0.5%)	0	1	1	0	0	1	0	0	1	0	1	0
12. Progressive retinal atrophy	3 (1.4%)	3	0	0	0	3	1	1	1	0	3	0	3
13. Proptosis of the eyeball	3 (1.4%)	1	2	0	3	0	1	2	0	2	1	3	0
14. Retinal detachment	3 (1.4%)	3	0	0	0	3	1	1	1	3	0	0	3
15. Traumatic injury	10 (4.8%)	4	6	6	2	2	3	5	2	3	7	6	4
16. Unilateral congenital anophthalmia	1 (0.5%)	0	1	0	1	0	1	0	0	0	1	1	0
Total	208 (100%)	130	78	91	54	63	69	74	65	106	102	74	134

* Eyelid problems include all the affection we found, such as entropion, ectropion, third-lid gland hyperplasia (cherry-eye), benign eyelid tumors like papilloma, and malign eyelids tumors like squamous cell carcinomas. ** Keratopathies included all corneal affection such as keratitis, corneal abrasions, corneal opacities (partial or total), corneal ulcers, and kerato-conjunctivitis.

**Figure 1:**
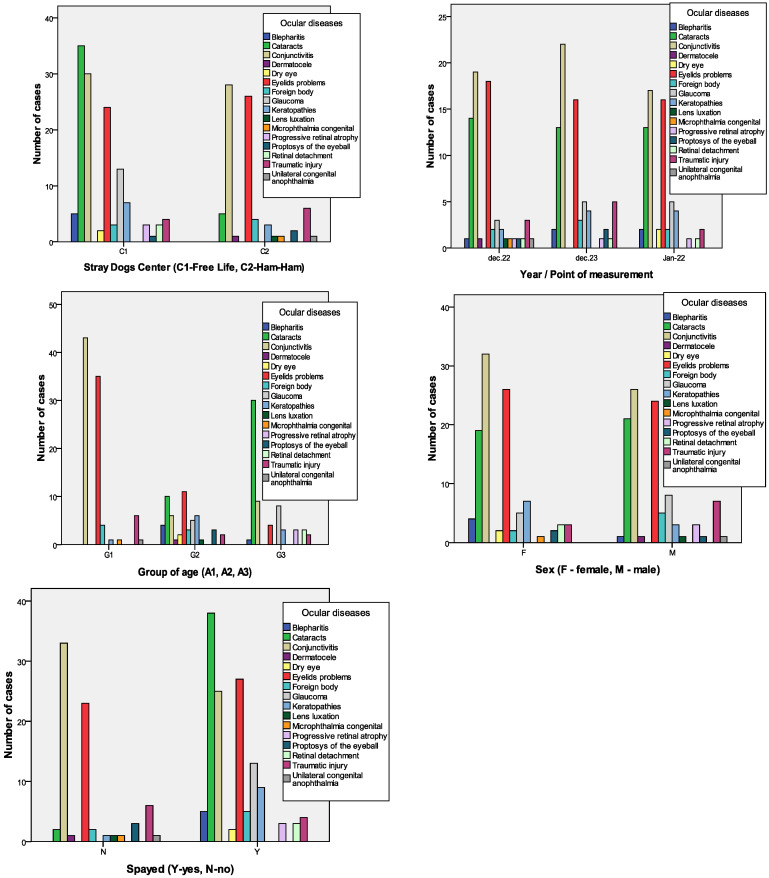
Bar charts of ocular disease prevalence stratifies for dogs’ centers, moment of measurements, age, sex, and castration status.

The most frequently encountered conditions were conjunctivitis (27.9%), eyelid problems (24%), and cataracts (19.2%). No significant associations were found between the year of study and the first three most prevalent eye affections: conjunctivitis (χ^2^ = 2.946, *p* = 0.229), eyelid problems (χ^2^ = 0.643, *p* = 0.725), or cataracts (χ^2^ = 0.414, *p* = 0.813). Details of the other ocular diseases are presented in Table 3 and illustrated in Figure 1.

**Figure 2:**
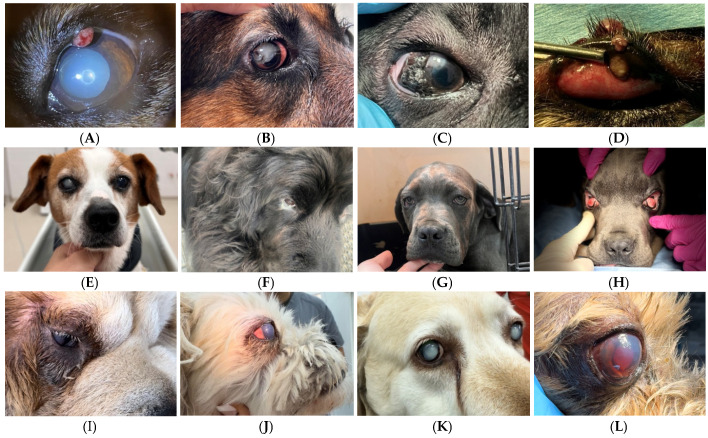
Ocular affections in dogs diagnosed during ocular examination in “Ham Ham”, “Free Life” Associations, and “Mobovet” Veterinary Clinic in Satu Mare, Romania. (**A**) cataract; (**B**) cataract-lens luxation; (**C**) dermoid; (**D**) eyelid papillomatosis; (**E**) traumatic cataract right eye; (**F**) purulent conjunctivitis; (**G**) bilateral ectropion; (**H**) hyperplasia of the third eyelid gland; (**I**) blepharitis; (**J**) glaucoma; (**K**) bilateral cataract and conjunctivitislid gland; (**L**) traumatic injury. **Source:** Original images from S.C. Mobovet SRL Veterinary Clinic, Satu Mare, collected between 2022 and 2023.

Generally, the ocular disease (Figure 2) was not associated with sex (female vs. male, χ^2^ = 20.043, *p* = 0.170) or the moments of measurement (P1, P2, and P3, χ^2^ = 17.933, *p* = 0.960). However, it was associated with the Stray Dogs Center (Free Life vs. Ham Ham, χ^2^ = 44.933, *p* < 0.000), age group (A1, A2, and A3, χ^2^ = 148.487, *p* < 0.000), and splayed class (yes vs. no, χ^2^ = 62.830, *p* < 0.000).

### 3.3. Prevalence of Ocular Diseases in Dogs by Age in Stray Dogs Centers

The dogs were classified into three age groups: puppies and young dogs (Group A1: 1 month to 2 years old, with 5.12 ± 0.42 months), adults (Group A2: 2 to 7 years old, with 48.11 ± 3.24 months), and aged dogs (Group A3: over 7 years old, with 113.62 ± 2.11 months).

During the study period, age appeared to be significantly associated with ocular problems (χ^2^ = 129.747, *p* < 0.000), with the youngest group (A1) showing the highest prevalence.The age of dogs in the “Free Life” Center was 67.80 ± 4.21 months, while in the “Ham Ham” Center, it was 18.08 ± 4.21 months.

The age difference of 49.72 months between the two centers was statistically significant (t = 8.232, *p*< 0.000). The total number of ocular problem cases was influenced by age groupings at both the “Free Life” (χ^2^ = 26.764, *p* < 0.000) and “Ham Ham” (χ^2^ = 245.154, *p* < 0.000) centers. Notably, Group A1 (dogs under 2 years old) had a higher percentage of ocular diseases, with 50.96% at the “Free Life” center and 65.51% at the “Ham Ham” center. A higher incidence was observed in aged dogs at the “Free Life” center (31.73%) compared to the “Ham Ham” center (20.68%) during the study period (Table 3).

The study identified significant associations between age groups and certain eye conditions. Conjunctivitis was more prevalent in the youngest age group (G1), with 43 out of 58 cases (χ^2^ = 11.945, *p* = 0.003). Younger dogs (A1 group) were 4.91 times more likely to develop conjunctivitis (OR = 4.91, 95% CI = 1.99–12.11, *p* = 0.001) compared to older dogs (A2 and A3 groups). Eyelid issues were more prevalent in younger dogs, representing 35 out of 50 cases (χ^2^ = 7.064, *p* = 0.029). Younger dogs were 9.38 times more likely to develop eyelid problems (OR = 9.38, 95% CI = 3.18–22.04, *p* < 0.000) compared to older dogs. While no significant association was found between age and cataracts in the stray centers (χ^2^ = 0.760, *p* = 0.783), a significant link was established between cataracts, age, and sex (χ^2^ = 5.647, *p* = 0.017). Younger dogs were 0.31 times less likely to develop cataracts (OR = 0.31, 95% CI = 0.12–0.83, *p* = 0.019) compared to older dogs. Additionally, cataract cases were significantly associated with castration status, with 38 out of 40 affected dogs being castrated (χ^2^ = 14.737, *p* < 0.000). Glaucoma also showed significant associations with both age and sex (χ^2^ = 13.000, *p* < 0.000), being more common in older dogs (G3 group).

### 3.4. Sterilization Status and Sex-Based Incidence

Out of the 208 dogs with ocular affections, 106 were female, and 102 were male. All dogs at both centers were spayed, and any new dogs entering the centers were sterilized as soon as possible. Older dogs (A2 and A3 groups) were 14.34 times more likely to have achieved sterilization status in the centers compared to younger (A1 group) ones (OR = 14.34, 95% CI = 5.06–40.64, *p* < 0.000). Some dogs already had ocular affections when they arrived and had not yet been sterilized. Our statistical analysis showed that spaying (Figure 2) significantly affects ocular conditions (χ^2^ = 62.830, *p* < 0.000), while no significant difference was found in the incidence of ocular conditions based on sex (frequencies: χ^2^ = 20.043, *p* = 0.170; odds ratio: OR = 1.02, 95% CI = 0.46–2.30, *p* = 0.953). The study found a significant association between the prevalence of conjunctivitis and both the age and reproductive status of the dogs. Specifically, conjunctivitis was more frequent in younger dogs (43 out of 48 cases in group A1, Pearson χ^2^ = 11.945, *p* = 0.003) and had a higher incidence in non-castrated dogs (33 out of 58 cases, χ^2^ = 4.661, *p* = 0.031). A similar pattern was observed for eyelid problems, the second most prevalent eye disease in the study. These issues were also more common in younger dogs (35 out of 50 cases, χ^2^ = 7.064, *p* = 0.029) and in castrated dogs (27 out of 50 cases, χ^2^ = 8.194, *p* = 0.004).

## 4. Discussion

In the literature, there is considerable variability in the prevalence of eye lesions observed in animal hospitals and stray dog centers, likely due to differences in the canine population and the clinical approach (generic or specialist) evaluated. The prevalence of ocular diseases in our study was 9.07%,which is higher than the findings of hospitals at 1.85% [17]. This variation may be attributed to geographical and environmental differences and local infrastructure conditions. Other authors [7,18] have noted that unfavorable weather conditions are associated with a higher incidence of ocular affections. Eye diseases reported in Europe, including Romania and Eastern Europe, are caused by various factors and etiological agents. For example, conjunctivitis can result from systemic conditions such as viral infections (e.g., canine distemper virus [9]), bacterial infections (e.g., *Borrelia burgdorferi* [19]), protozoal diseases (e.g., *Thelazia callipaeda* [11,20,21], *Leishmania infantum* [22,23,24]; *Acanth amoeba* [25]), and parasitic infections (e.g., *Onchocercalupi* [11,26]; *Thelazia callipaeda* [27]). Cataracts have been associated with systemic causes such as infectious diseases (e.g., Infectious canine hepatitis [10]), metabolic conditions (e.g., diabetes mellitus [14]; hyperadrenocorticism [13]), nutritional deficiencies (e.g., chronic Vitamin E deficiency [28]), and toxic exposures [15]. Retinal detachment has been linked to infectious diseases (e.g., *Borrelia burgdorferi* [19]), parasitic infections (e.g., *Dirofilaria immitis* [29]; *Dirofilaria repens* [30,31]), and cardiovascular conditions (e.g., systemic hypertension [32]).The same pattern is in South Europe. For example, in a municipal shelter in Sicily, Italy, the point prevalence of ocular lesions in 45 shelter dogs with leishmaniasis was 71.11% (45/127 dogs). The most frequent ocular lesion was blepharitis (50%), while anterior uveitis was observed in only 9.37% of cases [24]. Given these complex factors, Pennisi (2015) [33] emphasized that managing stray dogs and municipal kennels in endemic areas of Europe should be a priority for veterinary and public health authorities. Urgent implementation of effective preventive and therapeutic measures is necessary to address the high number of lifelong sheltered dogs in public kennels, where there is often a lack of adequate healthcare.

In our study, the higher prevalence may be due to the fact that many eye problems were diagnosed when the dogs were first brought to the shelters. Most stray dogs were collected from the streets, fields, and rural areas, where they had previously lived without any medical care and were exposed to harsh weather conditions such as rain, snow, and wind. Additionally, although the stray dogs in the two shelters have access to cages, they often remain exposed to the elements, which contribute to the development of conjunctivitis and other eye diseases. This difference may be attributed to the “Free Life” center being an older shelter with a higher percentage of old dogs that have not been adopted due to their health conditions (osteoarthritic, cardiac, or ophthalmologic problems) requiring lifelong treatment or because of their poor vision. Typically, most new arrivals at the shelter are puppies or dogs up to 4–5 years old, with older dogs rarely being admitted, usually due to owners who can no longer afford veterinary care to maintain their quality of life. Conjunctivitis, the most prevalent eye disease in the study, can be easily treated under normal conditions and often goes untreated until it results in more severe complications, including bacterial contamination [34].

The prevalence of common ocular diseases observed in our study aligns with findings from shelters in Sicily, Southern Italy [24]. In the study by Pietro et al., 2016 [24], the most frequently diagnosed conditions included blepharitis (52.54% of 31 out of 64 eyes from 32 dogs), conjunctivitis (40.68%), and periorbital alopecia (37.29%). Similarly, Soundarya et al., 2020 [6] reported conjunctivitis as the most prevalent ocular disorder in dogs at the Veterinary College Hospital in Bangalore, Southern India, accounting for 27.24% of cases, followed by glaucoma (7.86%) and cataract (11.79%). In Sale’s study (2004) [35] conducted in the surgery department, the anatomical categorization of ocular affections revealed that the majority of cases involved the lens (34%), followed by the cornea (28%), retina (11%), eyelid (9%), conjunctiva (8%), glaucoma (6%), and both the anterior chamber and globe (2% each). The categorization showed the highest incidence of cataracts (295 cases out of 868 ocular cases), followed by corneal ulcers (134 cases) and cherry eye (78 cases).

The study observations of glaucoma in stray dogs are consistent with the results of other authors [36,37]. Thus, Gelatt and Mackay (2004) [36] noted that most cases of glaucoma in purebred dogs occurred between the ages of 4 and 10 years. Similarly, Kato et al. (2006) [37] observed that the mean age of dogs with glaucoma was generally beyond middle age across nearly all breeds.

In our case, traumatic injuries accounted for 3.75%, and foreign bodies for 3.00% of ocular problems. Most foreign bodies in the eye were included under traumatic injuries, which may result from causes similar to those described by Tamilmahan et al. (2013) [7]. They noted that accidental injuries during play, violence, and traumatic foreign bodies contributed to 19.29% of all ocular problems in canines, a significantly higher percentage than what we observed. Different ocular diseases such as eye trauma, corneal opacity, conjunctivitis, corneal ulcers, keratitis, cataracts, blepharitis, entropion, and ectropion were identified as common ocular conditions in dogs by Kalaiselvan et al. (2009) [38]. Sale et al. (2013) [35] reported the highest incidence of cataracts, followed by corneal ulcers, cherry eye, and progressive retinal atrophy. Kumar et al. (2015) [4] reported that in pure breeds, pigmentary keratitis/keratoconjunctivitis (21.7%) and corneal ulcer/injury (21.7%) as the most common ocular disorders, followed by corneal opacity (18.3%) and epiphora (11.6%).

Concerning the prevalence of ocular diseases in dogs by age, Soundarya (2020) [6] observed that the occurrence of eye disorders in puppies (<7 months), young adults (7 months to 2 years), and older dogs (>7 years) was 33.70%, 17.41%, 28.65%, and 20.22%, respectively. This pattern is similar to our study data across all age groups. Specifically, the combined groups of puppies and young dogs in Soundarya’s study were comparable to our group of dogs under 2 years of age, which also had the highest occurrence of eye disorders. Other authors [3] found that the age distribution of ocular conditions showed the majority of cases (68.80%) occurred in animals less than 5 years of age. Older animals (>5 years) were less affected (6.93%). Middle-aged dogs (1–5 years) appeared more prone to eye affections compared to younger (<1 year) and older (>5 years) animals. The causes of ocular affections were unspecified in 60.61% of the cases audited, while 35.05% were due to trauma (such as snake bites, dog bites, cage injuries, and gunshot wounds), and 4.33% were attributed to systemic diseases [3]. Our findings contrast with those of Indian hospitals [7,8], which reported an increased incidence of ocular affections in dogs older than 5 years. In our study, the young group of dogs exhibited a higher percentage of ocular problems. Clinical examinations revealed that impaired vision significantly reduced the quality of life in shelter dogs, as those with eye issues ate less, some developed behavioral problems with other dogs, and those with total blindness were often underweight and frequently showed aggression toward other dogs and shelter staff.

In relation to sex-based incidence, the existing literature shows varied results: some reports indicate that a higher percentage of females (42.42%) are affected compared to males (35.49%) [3], while Sale (2013) [35] reported a higher incidence in males (60%).While many eye diseases are known to be hereditary, we found no studies directly linking the spayed status in dogs with ocular pathologies. Our study suggests that non-sterilized dogs are more susceptible to eye problems. We attribute this to behavioral issues influenced by sex hormones, such as aggression, territorial disputes, and competition for mates, which can lead to fights and subsequent injuries. The significant association between spaying and ocular affections observed in our study is intriguing and not easily explained by a direct physiological mechanism. One plausible explanation could relate to the conditions under which dogs arrive at the shelter. Many dogs enter the shelter already experiencing stress and health issues, including ocular affections, which may be exacerbated or unmasked during the initial period of adjustment and screening (ocular diagnostic).The data suggest that the association is likely reflective of the timing and conditions of the dogs’ arrival rather than a direct cause-and-effect relationship between spaying and ocular affections. Further research would be necessary to explore this observation in more depth and to clarify the potential underlying factors. In stray dog centers, the potential for herd diseases (HrD) similar to those seen in farm animals [39] can be identified. By applying the methodology used in HrD studies, it becomes possible to identify, quantify, and rank risk factors due to the similar technological conditions under which the animals are kept [39,40].

A limitation of the study was the lack of an etiological diagnosis for the eye disorders. However, even under these conditions, examining dogs upon admission and providing appropriate treatment significantly improves their quality of life and increases their chances of adoption; the dogs with poor or no visual acuity (mature cataract, retinal detachment, retinal progressive atrophy) were not adopted during the 2 years period. Overall, 87% of the adopted dogs were healthy, but among those with eye problems, the adoption rate was substantially higher for those that had been diagnosed and treated (91%) compared to those that had not received the ophthalmic examinations and treatment (9%). The relevance of our study lies in its focus on the unique challenges presented by the shelter environment, where dogs are often exposed to varying health risks, including ocular conditions that may otherwise be overlooked. Veterinary ophthalmologists [41] who work in or collaborate with shelters must be aware of the prevalence and types of eye disorders commonly found in stray dogs to provide timely and effective care.

## 5. Conclusions

Our study identifies a considerable prevalence of eye disorders among a large group of stray dogs that received comprehensive ophthalmic examinations in two stray dog centers in northwest Romania. The most affected dogs were both young and elderly, with a variety of eye conditions analyzed. The most common disorders included conjunctivitis, cataracts, eyelid problems, keratopathies, and traumatic injuries. By identifying the most common eye conditions and their prevalence in this specific population, our study highlights the importance of thorough ophthalmic examinations at the time of admission and appropriate treatment initiation. This is crucial not only for improving the dogs’ quality of life but also for increasing their chances of adoption, which is a key goal of shelter operations. Understanding these dynamics helps veterinarians better manage the health of shelter dogs, ultimately benefiting both the animals and the community.

## Figures and Tables

**Table 1:** The ophthalmic examination process.

Examination	Description of the Examination Process
1. Visual Assessment:	Determines if each eye is visual or not.
2. Eye Structure Inspection:	Checks if the eye structure is intact. Traumatic injuries, globe penetration, deep ulcers, or melting eyes can result in corneal tears.
3. Redness Pattern Inspection:	Identifies the pattern of redness and determines if it is due to subconjunctival hemorrhage, conjunctival hyperemia, ciliary flush, or a combination of these.
4. Conjunctival Discharge Assessment:	Notes the presence and categorize the discharge by amount (profuse or scant) and character (purulent, mucopurulent, serous, or hemorrhagic).
5. Cornea Examination:	Identifies surface problems or inflammation, ulcers (superficial or deep), opacities (including keratitic precipitates), and irregularities like corneal edema or leukoma. Uses a penlight for examination and fluorescein staining to check for corneal epithelium disruption.
6. Eye Position and Movement:	Assesses the position of the eyes and their movements in six cardinal positions if the globe is intact.
7. Pupil Examination:	Checks the pupils for size and reaction to light.
8. Lid and Adnexal Structures Examination:	Identifies modifications or injuries to the lids or other adnexal structures, including foreign bodies.
9. Intraocular Pressure (IOP) Measurement:	Performs tonometry to determine if the intraocular pressure is high, normal, or low, especially if glaucoma is suspected.
10. Ophthalmoscopy:	Uses a slit lamp biomicroscope to estimate the depth of the anterior chamber and detect microscopic blood (hyphema) or white blood cells (hypopyon), which indicate protein presence in the anterior chamber. Examines for lens modifications like cataracts, lens luxation, or retinal detachment.

## Data Availability

No new data were created or analyzed in this study. Data sharing is not applicable to this article.
